# Bayesian Inference for Mixed Model-Based Genome-Wide Analysis of Expression Quantitative Trait Loci by Gibbs Sampling

**DOI:** 10.3389/fgene.2019.00199

**Published:** 2019-03-22

**Authors:** Chaeyoung Lee

**Affiliations:** Department of Bioinformatics and Life Science, Soongsil University, Seoul, South Korea

**Keywords:** Markov chain Monte Carlo, expression quantitative trait locus, genetic association, Gibbs sampling, mixed model, polygenic variance component, posterior, random effect

## Abstract

The importance of expression quantitative trait locus (eQTL) has been emphasized in understanding the genetic basis of cellular activities and complex phenotypes. Mixed models can be employed to effectively identify eQTLs by explaining polygenic effects. In these mixed models, the polygenic effects are considered as random variables, and their variability is explained by the polygenic variance component. The polygenic and residual variance components are first estimated, and then eQTL effects are estimated depending on the variance component estimates within the frequentist mixed model framework. The Bayesian approach to the mixed model-based genome-wide eQTL analysis can also be applied to estimate the parameters that exhibit various benefits. Bayesian inferences on unknown parameters are based on their marginal posterior distributions, and the marginalization of the joint posterior distribution is a challenging task. This problem can be solved by employing a numerical algorithm of integrals called Gibbs sampling as a Markov chain Monte Carlo. This article reviews the mixed model-based Bayesian eQTL analysis by Gibbs sampling. Theoretical and practical issues of Bayesian inference are discussed using a concise description of Bayesian modeling and the corresponding Gibbs sampling. The strengths of Bayesian inference are also discussed. Posterior probability distribution in the Bayesian inference reflects uncertainty in unknown parameters. This factor is useful in the context of eQTL analysis where a sample size is too small to apply the frequentist approach. Bayesian inference based on the posterior that reflects prior knowledge, will be increasingly preferred with the accumulation of eQTL data. Extensive use of the mixed model-based Bayesian eQTL analysis will accelerate understanding of eQTLs exhibiting various regulatory functions.

## Introduction

**Figure 1:**
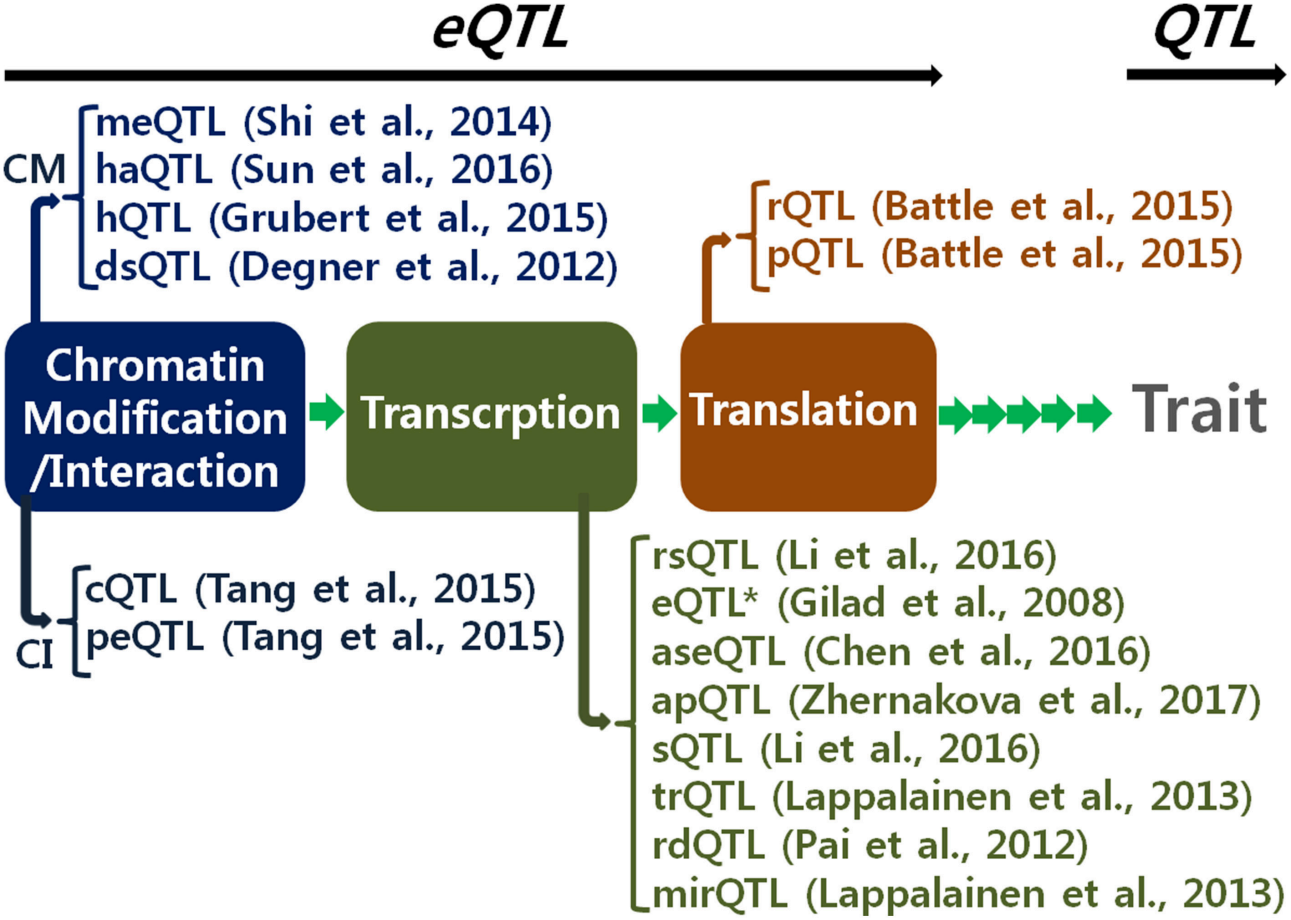
Various expression quantitative trait locus (eQTL) by regulatory stages. This allows fine resolution of eQTL as well as QTL (quantitative trait locus). CM, chromatin modification; CI, chromatin interaction; meQTL, methylation QTL; haQTL, histone acetylation QTL; hQTL, histone QTL; dsQTL, DNase I sensitivity QTL; cQTL, chromatin interaction QTL; peQTL, promoter enhancer interaction QTL; rsQTL, RNA synthesis rate QTL; eQTL*, narrow-sense eQTL; aseQTL, allele specific expression QTL; apQTL, alternative polyadenylation QTL; sQTL, splicing QTL; trQTL, transcript ratio QTL; rdQTL, RNA decay QTL; mirQTL, miRNA QTL; rQTL, ribosome occupancy QTL; pQRL, protein abundance QTL (Gilad et al., 2008; Degner et al., 2012; Pai et al., 2012; Shi et al., 2014; Battle et al., 2015; Grubert et al., 2015; Tang et al., 2015; Chen et al., 2016; Li et al., 2016; Sun et al., 2016; Zhernakova et al., 2017).

Identification of expression quantitative trait loci (eQTLs) is of great interest to geneticists studying the underlying genetic mechanisms of cellular activities and complex phenotypes. A genome-wide eQTL analysis makes it possible to determine a profile of regulatory signals for a single gene at a time. Recent technological developments have accelerated data production for genome-wide eQTL analysis. Research efforts have been made to obtain RNA-seq data to examine the profiles of eQTLs for all expressed genes in a single cell. For example, the Geuvadis consortium produced RNA-seq data using lymphoblastoid cell lines derived from 462 individuals participating in the 1,000 Genome Project (Lappalainen et al., 2013). More extensive RNA-seq data are available to examine spatial profiles of cells with different functions. The Genotype-Tissue Expression consortium produced spatial RNA-seq data using 1,641 samples taken across 43 tissues obtained from 175 individuals (GTEx Consortium, 2015). Additionally, data available on various expression molecules currently enables us to analyze regulatory stage-specific eQTLs as shown in Figure 1, to further understand specific regulatory functions of gene expression.

Gradual increases in such a delicate profile of spatial, temporal, and/or functional eQTLs requires a reasonable statistical inference. Mixed models have been employed to allow more accurate inferences from genome-wide association analyses, than conventional fixed models, which explain the genetic effect of only one candidate nucleotide variant and considers all other genetic effects as sampling errors (Kang et al., 2010; Zhang et al., 2010; Yang et al., 2011). As mixed models include polygenic effects as random effects, rather than as sampling errors, they can avoid spurious eQTLs produced by population stratification (Widmer et al., 2014; Shin and Lee, 2015). Population stratification is one of the most critical problems arising from such genome-wide association studies (Price et al., 2010). Although certain strategies such as genomic control (Devlin and Roeder, 1999) and principal component analysis (Price et al., 2006), for conventional analytical methods have been suggested to remove population stratification, these strategies are hardly satisfactory in overcoming this problem without considering the mixed model (Zhang et al., 2010; Ryoo and Lee, 2014). It is unrealistic to simultaneously include a number of individual nucleotide variant effects as fixed effects in conventional models in order to explain polygenic effects. This is because a large number of parameters for nucleotide variants cause critical problems, such as considerable reduction or lack of a degree of freedom and corresponding power. This may be reason enough to employ mixed models, even if these models possess no other strengths. Details concerning the strengths of using mixed models were discussed by Lee (2018).

Variability of the random polygenic effects is assessed as a polygenic variance component in mixed models. The eQTL effects are estimated depending on the polygenic and residual variance components estimated in a preliminary step. Thus, estimation of variance components is always stressed in mixed model methodology. A variety of methods to estimate variance components exist (Searle et al., 2009). Restricted maximum likelihood (REML) estimation is considered a standard method regardless of its computing algorithms in the frequentist mixed model framework (Lee, 2018).

Recently, the Bayesian approach has gained popularity and is increasingly used across many disciplines. The Bayesian approach is, however, rarely applied in the context of mixed model-based eQTL analysis. One likely reason for this is that the mixed model-based Bayesian inference is theoretically and computationally challenging. Currently, the burden of computation and memory has been greatly reduced by the development of advanced information technology. Algorithms for the practical application of the Bayesian approach are also available. Although a Bayesian approach for eQTL analysis was recently reviewed (Imprialou et al., 2017), the authors did not include the mixed model-based analysis. Thus, the present review should help geneticists to easily understand the background knowledge required for the Bayesian mixed model-based eQTL analysis and ultimately allow for the extensive use of this method. Additionally, the purpose of this review is to encourage those interested in developing relevant methods and algorithms for Bayesian inference. General concepts and considerations for genome-wide eQTL analysis using mixed models were discussed in the previous review (Lee, 2018). The current review highlights the Bayesian approach as a sequel to the frequentist approach for mixed model-based genome-wide eQTL analysis. The Bayesian analytical model, presented in a generalized form, is comparable to the frequentist model which has previously been reviewed. Minimal mathematical notations, to understand the Bayesian approach, are concisely presented without any intricacies of specific conditions. The definitions of statistical terms for the Bayesian mixed model-based eQTL analysis are summarized in Table 1.

**Table 1:** Summary of statistical terms for Bayesian mixed model-based eQTL analysis.

**Statistical term**	**Definition**
Statistical inference	Process of drawing conclusions about characteristics of a population in the presence of uncertainty using sample data
Bayesian inference	Statistical inference based on the posterior distribution of parameter reflecting both observed data and prior knowledge
Frequentist inference	Statistical inference based on only observed data
Parameter	Unknown numerical characteristic of a population
Statistic	Numerical characteristic of a sample
Prior	Probability distribution reflecting one's belief concerning a parameter
Posterior	Probability distribution of a parameter after taking into account the evidence obtained from observed data
Likelihood	Function of parameters given specific observed data
	The function has the same entity with another interpretation as a conditional density of the observed data given parameters
Mixed model	Analytical model including both fixed and random effects
	It is also called the mixed linear model, linear mixed model, or mixed-effect model
Fixed effect	Group -specific fixed quantity
Random effect	Subject-specific quantity considered as a random sample from a population
G-side modeling	Modeling repeated measures using random effects
R-side modeling	Modeling repeated measures using multiple residuals for each subject
Variance component	Parameter describing variability of random effects in the mixed model
Markov chain Monte Carlo	A numerical integration method for Monte Carlo generation of samples from a probability distribution updated by the Markov chain that leads parameters to converge to equilibrium distribution
Gibbs sampling	A Markov chain Monte Carlo method using all the full conditional probability distributions
Metropolis-Hastings algorithm	A Markov chain Monte Carlo method using approximate probability distributions due to difficulty in direct sampling from the distributions
Hamiltonian Monte Carlo	A Markov chain Monte Carlo method using approximate probability distributions, a Hamiltonian evolution between states, and targeting states with a larger acceptance criterion than observed probability

## Bayesian, Markov Chain Monte Carlo, and Gibbs Sampling

**Figure 2:**
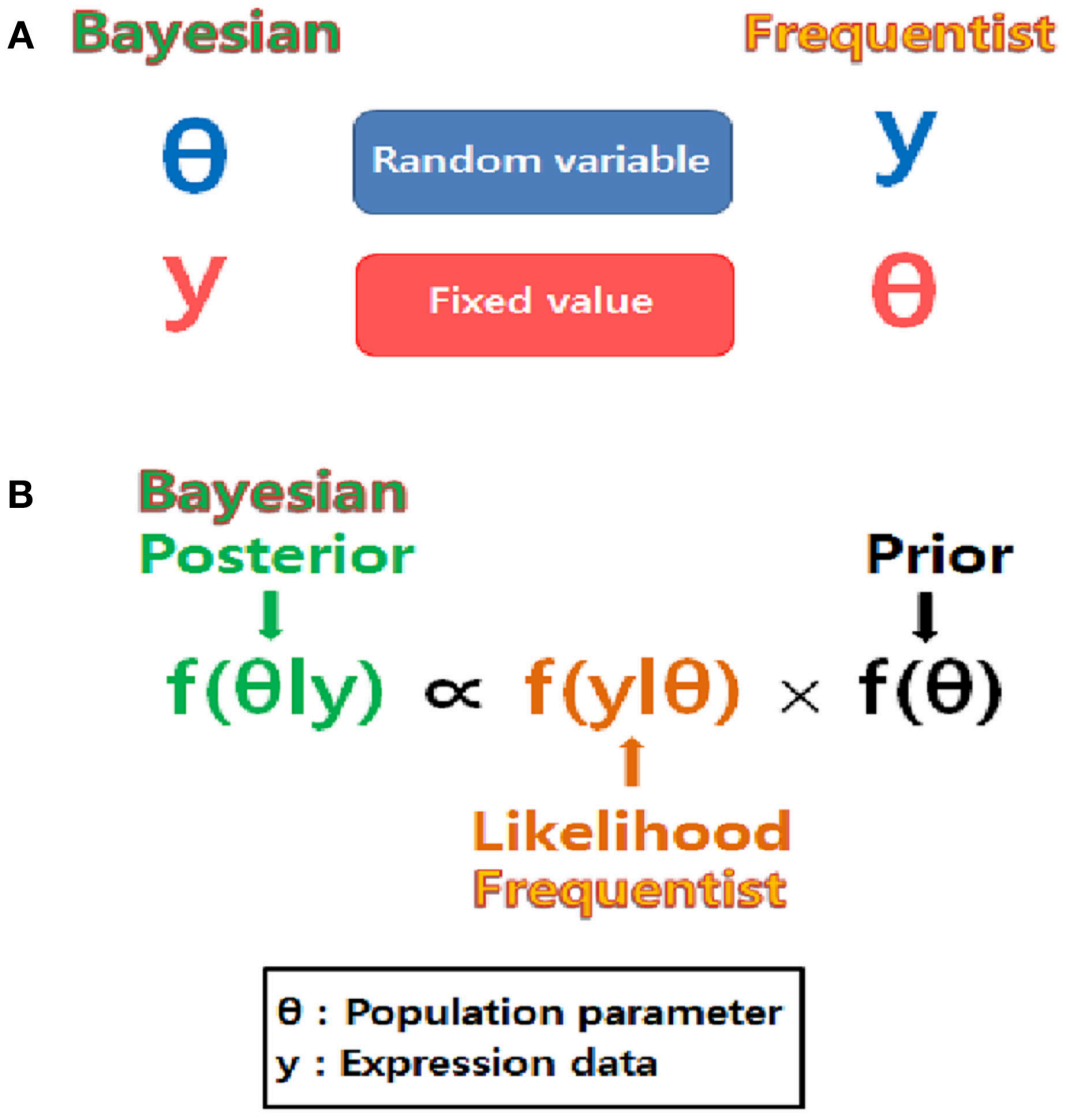
Different point of view between Bayesians and frequentists. **(A)** The nature of unknown parameters is compared. Parameters are considered as random variables in the Bayesian approach while they are considered as fixed values in the frequentist approach. **(B)** Bayesian inference is based on posterior distribution proportional to the product of likelihood and prior while frequentist inference is based only on likelihood.

Bayesian statistics reflect prior knowledge as well as observed data, while frequentist statistics depend only on observed data (Figure 2, Table 2). Bayesians also possess a different view than frequentists do, regarding the treatment of parameters (Figure 2). Bayesian inferences on unknown parameters are based on the marginal posterior distributions of these parameters. Thus, the Bayesian approach requires the integration-based elimination of nuisance parameters. When implementing the Bayesian approach, it is labor intensive to compute the integration of multi-dimensional functions to estimate the marginal posterior distributions. Marginalization of the joint posterior distribution can be attained through a variety of computational algorithms. A numerical algorithm of multi-dimensional integrals is the Markov chain Monte Carlo (MCMC; Tanner, 1993). A Monte Carlo integration generates independent samples, but the MCMC generates correlated samples through a Markov chain which provides an equilibrium distribution. In this review, Gibbs sampling is presented as an MCMC-based numerical integration method. Gibbs sampling requires a conditional distribution for every parameter to be sampled exactly (Gilks et al., 1995).

**Table 2:** Property of Bayesian and frequentist approach.

	**Bayesian**	**Frequentist**
Parameter	Random	Fixed
Inference	Based on posterior	Based on likelihood
Background knowledge	Yes	No
Representative algorithm	Gibbs sampling	Restricted maximum likelihood
Point estimation	Many point estimates from posterior (e.g., posterior mean, maximum a posteriori, posterior median)	One point estimate by a specific estimator (e.g., restricted maximum likelihood estimate)
Interval estimation	Credible interval	Confidence interval

## Mixed Models For Genome-Wide eQTL Analysis

A general form of the mixed model for genome-wide eQTL analyses can be briefly expressed with vectors and matrices as follows (Lee, 2018):

$$\begin{array}{l} \text{y}=X\beta +\text{g}+\varepsilon \end{array}$$

where **y** is the observation vector (*n* × 1) of gene expression levels; *n* is the number of the gene expression levels; **β** is the vector (*n*_*l*_ × 1) of fixed effects such as sex, age, and candidate nucleotide variant effects; *n*_*l*_ is the number of the fixed effects; **X** is the design matrix (*n* × *n*_*l*_) for the fixed effects; **g** is the vector (*n* × 1) of random polygenic effects; **ε** is the vector (*n* × 1) of random residuals. To identify eQTL, **β** includes the minor allele effect of the candidate single nucleotide variant, and the corresponding column of **X** includes elements of 0, 1, and 2 as the number of minor alleles under the assumption of an additive genetic model with a biallelic single nucleotide variant. The random variables **g** and **ε** in the analytical model have the following normal distributions:

$$\begin{array}{l} \text{g}\sim N\left(0,\text{A}\sigma_{g}^{2}\right)\\ \varepsilon \sim N\left(0,\text{I}\sigma_{\varepsilon }^{2}\right) \end{array}$$

where $\sigma_{g}^{2}$ is the polygenic variance component; $\sigma_{\varepsilon }^{2}$ is the residual variance component; **I** is the identity matrix (*n* × *n*); **A** is the genomic similarity matrix (*n* × *n*) with elements of pairwise genomic similarity coefficients based on genotypes of single nucleotide variants. The genomic similarity coefficient (*a*_*jk*_) between individuals *j* and *k* can be calculated as follows (Yang et al., 2011):

$$\begin{array}{l} a_{jk}=\frac{1}{n_{v}}\overset{n_{v}}{\underset{i=1}{\sum }}\frac{\left(\tau_{ij}-2f_{i}\right)\left(\tau_{ik}-2f_{i}\right)}{2f_{i}\left(1-f_{i}\right)} \end{array}$$

where *n*_*v*_ is the number of single nucleotide variants that contribute to the genomic similarity; τ_*ij*_ and τ_*ik*_ are the numbers (0, 1, or 2) of minor alleles for the single nucleotide variant *i* of the individuals *j* and *k*; *f*_*i*_ is the frequency of the minor allele.

## Bayesian eQTL Analysis Based on Mixed Models

Unlike fixed model analyses, the mixed model analyses for genome-wide eQTL mapping additionally includes random polygenic effects and the corresponding variance component, as shown above. Marginal posterior distribution is required for each unknown parameter in the Bayesian inference. Marginalization can be attained by using an MCMC-based numerical integration as mentioned above. This review presents a Gibbs sampler as an MCMC for mixed model-based Bayesian eQTL analysis.

The conditional density function of all parameters given gene expression levels is defined based on Bayes' theorem as follows:

$$\begin{array}{l} f\left(\beta ,\text{g},\sigma_{g}^{2},\sigma_{\varepsilon }^{2}|\text{y}\right)=\frac{f\left(\text{y}|\beta ,\text{g},\sigma_{g}^{2},\sigma_{\varepsilon }^{2}\right)f\left(\beta ,\text{g},\sigma_{g}^{2},\sigma_{\varepsilon }^{2}\right)}{f\left(\text{y}\right)} \end{array}$$

where *f* indicates function. Since the denominator *f*(**y**) is not a function of the parameters, the conditional density function is proportional to the numerator, i.e.,

$$\begin{array}{l} f\left(\beta ,\text{g},\sigma_{g}^{2},\sigma_{\varepsilon }^{2}|\text{y}\right)\propto f\left(\text{y}|\beta ,\text{g},\sigma_{g}^{2},\sigma_{\varepsilon }^{2}\right)f\left(\beta ,\text{g},\sigma_{g}^{2},\sigma_{\varepsilon }^{2}\right). \end{array}$$

The left-hand side is called posterior, and this is proportional to the product of the likelihood corresponding to $f(y|\beta ,g,\sigma_{g}^{2},\sigma_{\varepsilon }^{2})$ and the prior corresponding to $f\left(\beta ,\text{g},\sigma_{g}^{2},\sigma_{\varepsilon }^{2}\right)$ Since all the parameters are independent, except for **g**, which depends on the hyperparameter of $\sigma_{g}^{2}$ under the assumption of $\text{g}|\sigma_{g}^{2}\sim N\left(0,\text{A}\sigma_{g}^{2}\right)$ in the mixed model framework (i.e., $f\left(\text{g},\sigma_{g}^{2}\right)=f\left(\text{g}|\sigma_{g}^{2}\right)f\left(\sigma_{g}^{2}\right)$), the joint posterior can be expressed as follows:

$$\begin{array}{l} f\left(\beta ,\text{g},\sigma_{g}^{2},\sigma_{\varepsilon }^{2}|\text{y}\right)\propto f\left(\text{y}|\beta ,\text{g},\sigma_{\varepsilon }^{2}\right)f\left(\text{g}|\sigma_{g}^{2}\right)f\left(\beta \right)f\left(\sigma_{g}^{2}\right)f\left(\sigma_{\varepsilon }^{2}\right) \end{array}$$

Note that conditioning on the known genomic similarity matrix (**A**) is dropped in the formula to avoid confusion with parameters, i.e., $f\left(\text{g}|\sigma_{g}^{2}\right)$ is equivalently replaced with $f\left(\text{g}|\text{A},\sigma_{g}^{2}\right)$. Each component of the joint posterior can be assumed as follows. First, $f\left(\text{y}|\beta ,\text{g},\sigma_{\varepsilon }^{2}\right)$ is assumed to possess multivariate normal distribution as follows:

$$y|\beta ,g,\sigma_{\varepsilon }^{2}\sim \;N\left(X\beta +g,I\sigma_{\varepsilon }^{2}\right)$$

Second, $f\left(\text{g}|\sigma_{g}^{2}\right)$ is assumed to have multivariate normal distribution as explained above. The *f*(**β**) is assumed to have uniform distribution. The $f\left(\sigma_{g}^{2}\right)$ and $f\left(\sigma_{\varepsilon }^{2}\right)$ are assumed to possess scaled inverse chi-square distributions as conjugate priors.

Thus, the joint posterior density is presented as follows:

$$\begin{array}{l} f\left(\beta ,g,\sigma_{g}^{2},\sigma_{\varepsilon }^{2}|y\right)\propto \sigma_{\varepsilon }^{-n}\exp\left[-0.5\sigma_{\varepsilon }^{-2}{\left(y-X\beta -g\right)}^{\prime }\left(y-X\beta -g\right)\right]\\ \;\times \sigma_{g}^{-n}\exp\left(-0.5\sigma_{g}^{-2}g^{\prime }A^{-1}g\right)\times \sigma_{g}^{-\left(\kappa_{g}+2\right)}\exp\left(-0.5\sigma_{g}^{-2}\kappa_{g}\omega_{g}\right)\\ \;\times \sigma_{\varepsilon }^{-\left(\kappa_{\varepsilon }+2\right)}\exp\left(-0.5\sigma_{\varepsilon }^{-2}\kappa_{\varepsilon }\omega_{\varepsilon }\right) \end{array}$$

where κ_*g*_ and κ_**ε**_ are scale parameters of the scaled inverse chi-square distributions for $\pi \left(\sigma_{g}^{2}\right)$ and $\pi \left(\sigma_{\varepsilon }^{2}\right)$, and ω_*g*_ and ω_**ε**_ are shape parameters (degrees of freedom) of the distributions.

Full-conditional posterior density for each parameter is derived from the joint posterior density. Removing independent components of the parameter from the density function helps to determine the kernel of the full-conditional posterior density. As a result, full conditional density of a scalar solution of fixed and random effects exhibits the following Normal distribution:

(1) $$\begin{array}{r} {\text{s}}_{q}|{\text{s}}_{-q},\sigma_{g}^{2},\sigma_{\varepsilon }^{2},\text{y}\sim \;N\left({\text{c}}_{q,q}^{-1}\left({\text{r}}_{q}-{\text{c}}_{q,-q}{\text{s}}_{-q}\right),{\text{c}}_{q,q}^{-1}\right) \end{array}$$

where coefficient matrix $C=\left[\begin{array}{cc} {\text{c}}_{q,q} & c_{q,-q}\\ c_{-q,q} & C_{-q,-q} \end{array}\right]$, solution vector $s=\left[\begin{array}{c} {\text{s}}_{q}\\ s_{-q} \end{array}\right]$, and right-hand side vector $r=\left[\begin{array}{c} {\text{r}}_{q}\\ r_{-q} \end{array}\right]$ from the Henderson's mixed model equation (Henderson et al., 1959), i.e., **Cs** = **r** is equivalent to $\left[\begin{array}{cc} X^{\prime }X & X^{\prime }\\ X & I+\frac{\sigma_{\varepsilon }^{2}}{\sigma_{g}^{2}}A^{-1} \end{array}\right]\left[\begin{array}{c} \beta \\ g \end{array}\right]=\left[\begin{array}{c} X^{\prime }y\\ y \end{array}\right]$ (Lee, 2018).

The full conditional density of the polygenic variance component shows the following scaled inverse chi-square distribution:

(2) $$\begin{array}{r} \sigma_{g}^{2}|\text{s},\sigma_{\varepsilon }^{2},\text{y}\sim \;\chi_{s}^{-2}\left(n+\omega_{g},\;\omega_{g}\kappa_{g}+{\text{g}}^{\prime }\text{A}\text{g}\right) \end{array}$$

where $\chi_{s}^{-2}$ is the scaled inverse chi-square distribution. Similarly, the full conditional density of the residual variance component possesses the following scaled inverse chi-square distribution:

(3) $$\sigma_{\varepsilon }^{2}|s,\sigma_{g}^{2},y\sim \;\chi_{s}^{-2}\left[n+\omega_{\varepsilon },\;\omega_{\varepsilon }\kappa_{\varepsilon }+{\left(y-X\beta -g\right)}^{\prime }\left(y-X\beta -g\right)\right]$$

The Gibbs sampler requires intensive iterative sampling from the consecutively updated full conditional posterior distributions. Prior to the intensive iteration, arbitrary initial values are set for parameters. Each round of iteration in the Gibbs sampling, for example, consists of the following consecutive steps:

1. Sample individual fixed and random effects consecutively from the full conditional density of described by Equation (1).
2. Calculate residuals (**ε = y − Xβ − g**).
3. Calculate quadratics for polygenic effects.
4. Sample the polygenic variance component from the full conditional density as described in Equation (2).
5. Calculate quadratics for residuals.
6. Sample the residual variance component from the full conditional density as described in Equation (3).

Burn-in periods and thinning intervals are determined to collect samples for posterior distributions of parameters. Samples generated until the Gibbs chain approaches a stationary distribution are all discarded as a burn-in period. Saving only every *m*^th^ sample after the burn-in period functions to reduce lag correlation among samples produced by the Markov chain and thus avoids sample size inflation. The *m* is termed the “thinning interval” for the Gibbs chain.

## Considerations and Cautions For Bayesian eQTL Analyses Using Mixed Models

A key advantage of the Bayesian approach is that it applies prior knowledge to statistical inference. When prior knowledge on the parameters is unavailable in a Bayesian approach, uninformative or flat prior is intuitively considered. Such an application should be used with caution, as it may lead to a theoretically improper prior (Hobert and Casella, 1996). For example, a flat prior assumed for polygenic or residual variance component can produce an undesirable situation where the integral of posterior probability converges to one without any convergence of prior integral. In this case, a weakly informative prior may be applied by employing a small value of the degree of belief hyperparameter (ω_*g*_) of the prior distribution for the variance component. This avoids the improper prior, and the resulting posterior distribution largely reflects the likelihood in practice.

Prior knowledge is increasing as eQTL data accumulates. However, a careful application of this knowledge, considering genetic covariance between populations and/or tissues, is required to maintain assumptions in practice. In addition, examining robust variable selections are needed to predict gene expression as eQTL data accumulates (Wu and Ma, 2015).

The burn-in period of the Gibbs chain may increase considerably if a poor choice of initial values occurs. It is particularly important to use initial values of fixed and random effects solved using the Henderson's mixed model equation with arbitrary initial values of variance components. The equation can be solved without the heavy computational burden of inversion of its huge coefficient matrix (**C**) by using either the Gauss-Seidel iteration (Van Tassell and Van Vleck, 1996; Lee and Pollak, 2002) or the Cholesky decomposition (Lee, 2016). Computing costs can be reduced by Hamiltonian dynamics, employing a Hamiltonian evolution between successive states and targeting states with a larger acceptance criterion than observed probability (Table 1; Girolami and Calderhead, 2011). This method reduces autocorrelation between samples and thus reduces computing cost for the post burn-in period as well as the burn-in period. Gibbs sampling may begin with optimal initial values to reduce the computing cost for the burn-in period. Restricted maximum likelihood (REML) estimates or their proximity are often considered as the initial values of variance components. This often helps avoid an undesirable situation where a Gibbs chain remains for a long time within a misplaced local region of the parameter space. This situation is generally accompanied by multimodal distributions. A preemptive way to avoid the multimodal situation is to run multiple Gibbs chains using different initial values (Gelman and Rubin, 1992).

Determination of the convergence where the Gibbs chain has reached the stationary distribution can be achieved using the Raftery-Lewis diagnostic (Raftery and Lewis, 1992). The diagnostic is based on the criterion of accuracy of a posterior quantile estimate. For each parameter, one can calculate the minimum number of iterations needed to estimate the quantile within a certain level of accuracy. The maximum for these values is determined as the number of iterations. This method can be used as any run length control diagnostic of the Gibbs chain. Specifically, the Raftery-Lewis diagnostic determines the burn-in period and also the thinning rate and the minimum post burn-in length of the Gibbs chain.

Various Bayesian point estimators are feasible from the posterior distribution (Table 2). The posterior mean is most commonly used as a Bayesian point estimator to minimize the risk function for a quadratic error loss. The posterior mean of each parameter is estimated not by averaging the sampled values, but instead by averaging the conditional expected values of the parameter to minimize variance. The expected values are not always available, however, and if they are absent then the posterior mean is obtained using observed values (Van Tassell and Van Vleck, 1996). For example, the posterior mean for the polygenic variance component can be calculated using expected values, while the posterior mean for heritability is calculated using observed values.

Parameter identifiability is a great concern to Bayesians. Identifiability of the polygenic effects depends on degree of similarity between individuals using nucleotide variants selected over the entire genome. It is possible for the parameters to have weak- or non-identifiability, which might be produced by analyzing gene expression regulated by a limited number of nucleotide variants or by using the similarity coefficients calculated with a large portion of undesirable nucleotide variants with spurious association (Ryoo and Lee, 2014). Although a posterior mean larger than zero for polygenic variance component is estimated, some diagnostics are suggested for the identifiability. An example for determining the identifiability is to examine whether the maximum region of posterior probability is localized or extends over a significant portion of the posterior range (Hines et al., 2014). Another example is to compare posterior distributions of the parameters to their corresponding prior distributions. Since the distributions are likely to be similar with a weak identifiability, percent overlap between the prior and posterior distributions might be used to assess the identifiability (Garrett and Zeger, 2000).

Simultaneous analysis of eQTL for two or more genes can be extended to the analysis described here. The major difference between simultaneous and separate analyses is the presence or absence of polygenic covariance component that explains polygenic effects shared by the expression of two genes. The prior for polygenic variance and covariance components is assumed to possess inverse Wishart distribution, which corresponds to the scaled inverse chi-square distribution under the assumption that the polygenic covariance component is equal to zero (Van Tassell and Van Vleck, 1996; Lee and Pollak, 2002). Although the polygenic covariance component estimates are obtained by simultaneous analysis, computing cost dramatically increases as the number of genes increases. Separate analysis requires arithmetically increased cost as the number of genes increases, however, simultaneous analysis requires exponentially increased cost. Simultaneous analysis with a large number of parameters is also likely to increase problems with convergence to target distribution. Thus, simultaneous analysis of eQTL for a small number of genes is recommended in practice. In fact, polygenic covariance component estimates of interest are all obtained by pairwise simultaneous analysis (i.e., eQTL analysis for two genes at once). Such a simultaneous analysis of eQTL can also be applied to identify a variety of temporal (e.g., day and night by circadian rhythm) and spatial (e.g., multiple tissues) eQTLs for a specific gene.

The current review focuses on Gibbs sampling as the most widely used MCMC algorithm to obtain random samples from a probability distribution, for which direct sampling is difficult in the mixed model-based Bayesian eQTL analysis. However, other MCMC algorithms can also be considered. The Metropolis-Hastings method might be employed to draw samples when the posterior for a certain variable does not have any kernel fit a standard density form (Hastings, 1970). A candidate sample is generated from a proposal distribution, and then acceptance or rejection of the candidate sample is determined according to a rule based on proposal distribution and desired distribution. The accepted candidate value is used in the next iteration. The rejected candidate value is discarded, and current value is reused in the next iteration. Hamiltonian Monte Carlo is another MCMC algorithm to approximate probability distributions. This algorithm employs a Hamiltonian evolution and targets states using a larger acceptance criterion than observed probability (Duane et al., 1987; Neal, 2011). This leads to a reduction of autocorrelation between consecutive samples and of course a quick convergence of the stationary distribution.

## Strengths of Bayesian eQTL analyses Using Mixed Models

The strengths of employing the mixed model analysis of eQTLs were intensively discussed in the previous review (Lee, 2018). They are, of course, all valid in the Bayesian approach incorporated with the mixed model. Thus, this section will focus on advantages that the Bayesian approach affords, compared to the frequentist approach.

The Bayesian approach, implemented with Gibbs sampling, provides empirical Bayes estimates of fixed effects and random effects, which correspond to the best linear unbiased estimator (BLUE) and the best linear unbiased predictor (BLUP), respectively. Sample-based estimates of polygenic and residual variance components, instead of unknown variance components, are used at every round of the Gibbs chain. Conversely, the frequentist approach first to estimates the variance components and then estimates the fixed and random effects, based on the variance component estimates. This produces a problem of non-BLUE and non-BLUP conditions by violating the assumption of known variance components required for BLUE and BLUP (Lee, 2018). Furthermore, no penalty is imposed for the use of variance component estimates instead of true values when the fixed and random effects are estimated. As a result, the frequentist cannot explain error variability inflated by replacement with variance component estimates.

Uncertainty in the unknown polygenic and residual variance components is reflected in the Bayesian analysis, by treating the unknown parameters as random variables. The Bayesian analysis results in a probability distribution (i.e., posterior) for each parameter. This enables us to make straightforward inferences concerning the parameters. For example, specific credible intervals for every parameter can be directly obtained using the samples generated from the posterior distribution by the Gibbs sampler. This credible interval is interpreted as a range within which a parameter value falls, with a specific probability. This is intuitively more acceptable than the confidence interval, with which frequentists interpret the confidence level as the proportion of the confidence intervals that contain the true value of parameter when confidence intervals are repeatedly estimated from independent sample statistics. Of course, confidence intervals are not repeatedly estimated in reality. As uncertainty is also reflected by the probability distribution, the Bayesian analysis does not require large samples. Given this, the resulting posterior allows for the calculation of probability of the true parameter (e.g., polygenic variance component) although the posterior might show the dispersed distribution with a large variance. Conversely, a large amount of data is necessary as the core assumption for the frequentist approach (Casella and Berger, 1990).

## Software

Bayesian analysis is mathematically and computationally demanding, making it difficult to put into practice. Useful software is, however, available to efficiently apply the Bayesian approach to a mixed model-based genome-wide eQTL analysis (Table 3). The Gibbs sampling described in this article has been implemented with the software of OpenBUGS (Lunn et al., 2009), GENSEL (Kizilkaya et al., 2010), MTGSAM (Van Tassell and Van Vleck, 1996), and rjags (Plummer, 2018). In particular, the OpenBUGS runs with Windows and Linux as the open source version originated from WinBUGS, one of the most popular programs used to fit Bayesian analysis by Gibbs sampling (Lunn et al., 2000). The rjags provides a user interface from R to the Just Another Gibbs Sampler (JAGS). This R package allows the use of a program provided by the OpenBUGS and can also easily program a user's own algorithms with different functions, distributions, and/or samplers (Plummer, 2018). Since the GENSEL was originally developed for whole genome prediction and genomic selection in animal and plant breeding, the program was devised to deal more efficiently with individual polygenic effects with regard to genomic selection compared to OpenBUGS or rjags. The MTGSAM can produce a genetic relationship matrix using pedigree information and efficiently deal with the matrix and its inverse, which are sparse (Van Tassell and Van Vleck, 1996). This is useful for explaining polygenic effects of closely related animals, which are often produced by artificial insemination and embryo transfer.

**Table 3:** Useful software for Bayesian genome-wide eQTL analysis using mixed models.

**Program**	**Method^a^**	**Website (http)**	**MA^b^**	**Source code**	**References**
OpenBUGS	Gibbs sampling	www.mrc-bsu.cam.ac.uk/software/bugs	O	Component Pascal	Lunn et al., 2009
MTGSAM	Gibbs sampling	aipl.arsusda.gov/software/mtgsam	O	Fortran	Van Tassell and Van Vleck, 1996
GENSEL	Gibbs sampling	archive.is/bigs.ansci.iastate.edu	X	C++	Kizilkaya et al., 2010
rjags	Gibbs sampling	mcmc-jags.sourceforge.net	O	R	Plummer, 2018
GEMMA	Metropolis-Hastings	www.xzlab.org/software.html	O	C++	Zhou et al., 2013
Stan	Hamiltonian Monte Carlo	mc-stan.org	O	C++	Carpenter et al., 2017
Gibbsit	Raftery-Lewis diagnostic	lib.stat.cmu.edu/general/gibbsit	NA	Fortran	Raftery and Lewis, 1992

a Markov chain Monte Carlo methods for generating random samples from a probability distribution. The Raftery-Lewis diagnostic is a method for controlling length of the Gibbs chain.

b *Multivariate analysis*.

GEMMA and Stan employ other MCMC algorithms to implement Bayesian analysis. The GEMMA implements the Metropolis-Hastings algorithm to estimate the proportion of total variance explained by the candidate eQTL and polygenic effects (Zhou et al., 2013). The Stan implements the Hamiltonian Monte Carlo sampling algorithm and provides the user with interfaces of CmdStan for the command line shell, RStan for R, and PyStan for Python (Carpenter et al., 2017). The Raftery-Lewis diagnostic method to control the Gibbs chain length has been implemented with the Gibbsit program (Raftery and Lewis, 1992).

## Computational Challenge

Although algorithms and software are available for application to the mixed model-based Bayesian eQTL analysis, researchers are confronted with a problem of computational costs in practice of the Bayesian analysis, which requires intensive computing. It is quite expensive and greatly dependent on the numbers of subjects, loci, and genes. For example, approximately a month of computation time is required for the Bayesian estimates with 100 subjects, 200,000 loci, and 5,000 genes in transcriptome- and genome-wide association analyses using a desktop processor (Intel® Core™ i7-8700K Processor; 4.7 GHz, 64 GB DDR4).

Some strategies might be useful to reduce computation time. First, computational burden is reduced by limiting the amount of analyses. After a preliminary study, only a limited number of genes and/or loci can be subsequently examined by the Bayesian analysis. For example, Bayesian inference might be applied to a genome-wide eQTL analysis, with the candidate genes identified by transcriptome-wide association analysis, or to regional (e.g., *cis*-eQTL) and/or candidate eQTL analysis. Furthermore, only a representative variant within each linkage disequilibrium block can be considered for identifying eQTLs. Second, reduction in computational burden can be achieved by employing efficient algorithms. For example, use of Hamiltonian Monte Carlo can reduce the number of iterations, by decreasing autocorrelation between successive samples, as explained above. Third, parallel computation is important to reduce computation time. Analyses by individual candidate genes or by their groups can be carried out simultaneously. This also helps reduce computing time in solving a gene- or eQTL-specific problem. Lastly, computation time is reduced by high performance computing facilities. In particular, cloud computing provides efficient techniques for intensive parallel computing (Hamdaqa and Tahvildari, 2012). If an efficient parallel computation facility (i.e., multiple processors) is used for the Bayesian analysis with the reduced number of genes from 5,000 to 50, it is possible to complete the analyses in a few hours.

## Closing Remarks

This review is provided for geneticists to understand the various backgrounds of mixed model-based Bayesian eQTL mapping. This may aid geneticists to overcome their skepticism of the Bayesian approach. As small or even negligible differences are often observed in practice between estimates resulting from the Bayesian approach and the frequentist approach, geneticists tend to possess a neutral point of view concerning these approaches, and they are reluctant to employ the Bayesian method simply because of its difficulty in theory and computation. As explained in this review, the advantages of the Bayesian approach are considerable and can be applied to the mixed model-based eQTL analysis. In particular, the inference on probability distribution of parameters in the Bayesian approach, provides a major advantage by reflecting uncertainty in unknown parameters. In comparison, the frequentist approach requires a large number of samples to estimate the true parameter as a critical assumption. Sample size for genome-wide eQTL analysis is not usually large, particularly in comparison to those of genome-wide association analyses of complex phenotypes. The prior is becoming important, as systems genetics improves in conjunction with a dramatic increase of eQTL data in the near future. The Bayesian approach will considerably aid researchers to examine eQTLs and understand their regulatory functions by characterizing eQTLs using various techniques.

## Author Contributions

The author confirms being the sole contributor of this work and has approved it for publication.

### Conflict of Interest Statement

The author declares that the research was conducted in the absence of any commercial or financial relationships that could be construed as a potential conflict of interest.
